# Social work practice and outcomes in rehabilitation: a scoping review

**DOI:** 10.3389/fresc.2024.1348294

**Published:** 2024-11-18

**Authors:** Nadja Freymüller, Tobias Knoop, Thorsten Meyer-Feil

**Affiliations:** Institute for Rehabilitation Medicine, Martin-Luther-University Halle-Wittenberg, Halle (Saale), Germany

**Keywords:** social work, rehabilitation professionals, rehabilitation team, rehabilitation outcome, scoping review

## Abstract

Social work is a long-established profession in health care and rehabilitation. Reviewing the evidence on effects of social work interventions shows inconsistencies, with several studies indicating positive, negative, or no significant effect at all. Against this background, the purpose of this paper is to provide an international overview of the research on social work practice in rehabilitation. Two research questions about the activities performed by social workers in rehabilitation settings and the reported outcomes to evaluate social work interventions were guiding the analysis. A scoping review was conducted in order to identify these activities and reported outcomes. The literature search was carried out in two databases (PubMed, SocINDEX). Additionally, the authors searched manually for literature in rehabilitation science and social work journals. Inclusion criteria encompassed the involvement of social workers and a description of their activities. The context in which social work's practice had to take place was a rehabilitation setting. A total of 2,681 records could be identified by searching the databases, journals, proceedings and reference lists. 66 sources met the predefined inclusion criteria. A majority of the identified activities that social workers perform are case related. Topics that may occur in these case encounters are the social environment of the patient, financial/social security, work-related issues and others. Of particular note are activities such as assessment, counseling and education. When applying the ICF framework, the outcomes are distributed across almost all components with an emphasis on Participation. This review demonstrates that social work has a vital role in the interprofessional rehabilitation team on an international level. However, there is still a need for more research about the effectiveness of social work interventions. We identified internationally common social work core activities/issues and derived a proposal for specific outcomes for future evaluation research.

## Introduction

Social work is a long-established profession in health care (1, 2). There are a variety of terms used to describe social workers, such as medical social workers, health social workers or clinical social workers (3). In a health care setting, social work addresses people with health conditions who have difficulties in living independently or people who are likely to be impaired in their social and/or vocational participation. Social workers are part of an interprofessional team and address the psychosocial needs of patients while taking a bio-psycho-social perspective (4, 5). They carry out a bundle of complex interventions which are characterized by a variety of components, target groups and settings as well as by a high level of flexibility (6).

In the field of rehabilitation, social workers may also be found as members of the interprofessional rehabilitation team. Early references to social work in connection with rehabilitation can be traced back to the 1950s in the United States (7, 8). Abrams and Dana juxtaposed social work and vocational counseling due to their overlapping areas of responsibility in the field of rehabilitation. They emphasized social work's resource-oriented approach and holistic perspective on the patient and pointed out activities of the profession in the rehabilitation process, such as psychosocial assessments, treatment on an individual, group or community level as well as activating community resources (7). Wallace explored the responsibilities of social workers in rehabilitation, specifically highlighting their roles in providing information and counseling as well as actively participating in the rehabilitation team. In addition, she emphasized the importance of considering patients’ social needs, thereby employing a holistic perspective (8).

Recent literature refers to psychosocial assessments, counseling and health education, discharge planning, case management, and involving different stakeholders as purviews of social work in rehabilitation (9, 10). To date, this literature offers no consistent or specific description of possible outcomes of social work interventions in rehabilitation (9, 11). A review revealed that social work practice can vary across rehabilitation facilities and indications, although the articles reviewed predominantly originated from Germany (12). Nevertheless, a plurality of indications and different rehabilitation settings suggest potential variation in practice on an international level as well.

Reviewing the evidence on social work interventions shows inconsistencies, with several studies indicating positive, negative, or no significant effect at all. In addition, the trials cover a wide array of outcomes, which makes comparability of the studies difficult (12, 13). This impedes social worker decision-making regarding evidence-based practice (EBP), which is defined here as the selection of interventions under consideration of the best research evidence, clinical expertise and patient preferences (14, 15).

A recent study (“Causal Assumptions about Social Work Services in Medical Rehabilitation”) ties in with the findings of the aforementioned review and concerns the practice of social work and the development of a program theory for social work in medical rehabilitation in Germany (16). Findings encompassed the indication of a varying practice in terms of, e.g., the degree of coproduction of social workers, the handling of professional mandates, social work's involvement or its roles in the rehabilitation team (17, 18). The latter findings align with research on social work in interprofessional health care settings, which suggests that some team members may lack adequate understanding of the social worker's professional role, leading to inappropriate task assignments or underutilization of the full range of professional skills (19, 20).

Against this background, the purpose of this scoping review is to provide an international overview of the research on social work practice in rehabilitation. To our knowledge, this marks the first review dealing with this topic. While other reviews have considered social work practice, they have either focused on other health care settings (21, 22) or lacked comprehensive coverage on an international level (12). Therefore, the goal of this review is to globally map social work activities in rehabilitation and to analyze reported outcomes from studies on social work interventions. The following research questions were guiding: (1) Which activities are performed by social workers in rehabilitation settings? (2) What outcomes are reported to evaluate social work interventions?

## Methods

A scoping review was conducted following the guidance document for conducting scoping reviews by the Joanna Briggs Institute (JBI) (23) and the Preferred Reporting Items for Systematic reviews and Meta-Analyses extension for Scoping Reviews (PRISMA-ScR) (24). A protocol was registered at the platform of the Open Science Framework (25).

### Information sources and search strategy

The literature search was carried out in two databases (PubMed, SocINDEX). Search strategies were developed by combining and using subject headings of social work and rehabilitation as well as related terms. Table S1 of the Supplementary Material provides the search strategies for both databases. Furthermore, a thorough manual search was performed. The authors chose to conduct a comprehensive manual search in addition to a database search based on the experience made during the earlier mentioned review on social work practice which found the manual search to be more effective in terms of included studies (12). The authors searched for literature in rehabilitation science and social work journals. The selection of the former, was based on the Journal Citation Reports. Journals were filtered by using the category Rehabilitation. From the top 100 ranked journals, we selected those whose titles potentially relate to the research question. Journals with a focus on physiotherapy, for example, were not included in the shortlist. The social work journals were selected based on a ranking on disciplinary journals by Hodge et al. (26). In addition, the journal Social Work in Disability & Rehabilitation, which was discontinued in 2017, was searched due to its high relevance to the research questions. In total, the manual search comprised 18 rehabilitation science and 32 social work journals. Rehabilitation science journals were searched by using the term “social work” and vice versa, social work journals were searched using the term “rehabilitation”. If the search settings of the journals allowed it, only abstracts were searched for these terms. A complete list of the searched journals is attached (Table S2 Supplementary Material). Apart from the manual search in disciplinary journals, another manual search in rehabilitation congress proceedings was performed. The list of the searched congress proceedings can be found in Table S3 of the Supplementary Material as well.

### Study selection and eligibility criteria

In September 2022 we conducted the initial search which was updated in September 2023. The search was followed by an independent title and abstract screening by two reviewers (1st and 2nd author). All disagreements could be resolved by consensus. The same two reviewers independently carried out a subsequent full-text screening, resolving disagreements again by consensus. Reference lists of identified reviews were also searched for literature that met the inclusion criteria. No authors were contacted regarding the identification of additional literature.

Title-, abstract- and full-text screening were guided by the eligibility criteria established *a priori* that defined the population, concepts and context as recommended by Peters et al. (23). The authors included sources in which the involvement of social workers was described. In case other professions, such as case managers, were mentioned, a social work background of the respective professionals had to be explicitly stated in the source for it to be considered. Additionally, the target group/patients had to be over 18 years old. Patients with substance use disorders were excluded due to the fact that the standard of rehabilitative care may strongly differ compared to other indications (Population). Since the main objective of the scoping review was to map the practice of social work in rehabilitation at an international level, social work's practice represents a key concept. Hence, the mere reference of social work was insufficient for inclusion of full-texts. The sources, instead, needed to describe the activities performed by social workers, types of interprofessional cooperation, or interventions conducted by social workers. In the case of the latter, the reported outcomes portrayed an additional concept (Concepts). The context in which social work's practice had to take place was a rehabilitation setting. For defining such a setting, the definition of Negrini et al. (27) and the broader definition of the German Association for Rehabilitation (Deutsche Vereinigung für Rehabilitation) (28, see 29 for an English translation) was consulted. No restrictions were made concerning geographic location, since an international overview should be given (Context). Publications in English and German language that were published after 2010 were included as well as all types of evidence sources.

### Data extraction and charting

The two authors who conducted the screening of the identified records extracted and synthesized the data. Along with citation details (authors, date, title, journal), the country and the medical indication of patients were extracted. For the latter, a rather high abstraction level was chosen, e.g., carcinoses were grouped under oncology, but without further differentiation by type of tumor. Solely, neurological disorders were further differentiated, as a majority of included sources referred to neurological patients. Thus, traumatic (TBI) and acquired brain injuries (ABI), stroke and spinal cord injuries (SCI) were denoted. In addition, publications that focus on individuals receiving rehabilitation services due to the burden and role strain as a result of caregiving were categorized under caregiver burden. Concerning the rehabilitation setting, the authors roughly distinguished between medical and vocational rehabilitation services. Medical rehabilitation services were further differentiated between inpatient and outpatient services as well as services that took place in the home environment of patients, in a community setting or that were carried out digitally/*via* telephone. Furthermore, the study design of publications was extracted. The groupings included randomized controlled trials (RCT), non-randomized studies on interventions (NRSI), single arm pre-/post design, observational studies, feasibility studies, qualitative studies, mixed-methods designs and reports.

With regard to social work activities, a rough discrimination was made between publications focusing solely on social work as a profession and publications also involving other rehabilitation professionals. We screened the included records for descriptions of social work activities and outcomes of social work interventions and transferred them in their entirety to an extraction table. Afterwards, we entered the respective document into MAXQDA, a computer assisted software for qualitative data analysis (30). Guided by the recommendations for the extraction, analysis, and presentation of results in scoping reviews by Pollock et al. (31) a thematic coding approach (32) was used to abstract and map the data. During the preparation phase of the analysis, it was decided to apply an inductive as well as a deductive approach. More specifically, social work activities were extracted and analyzed using inductive coding and reported outcomes by using a deductive approach. Outcomes were extracted solely from studies that focused on interventions that were carried out by social workers. The identified outcomes were eventually linked to the components of the International Classification of Functioning, Disability and Health (ICF) (33). Social work activities were extracted from all identified sources. Open coding was initially conducted, and the resulting codes were organized and summarized as needed (e.g., if they were too similar). This led to the creation of a framework with two levels of categories, namely main and subcategories. In a final step, the data was revisited and assigned to the framework.

## Results

**Figure 1:**
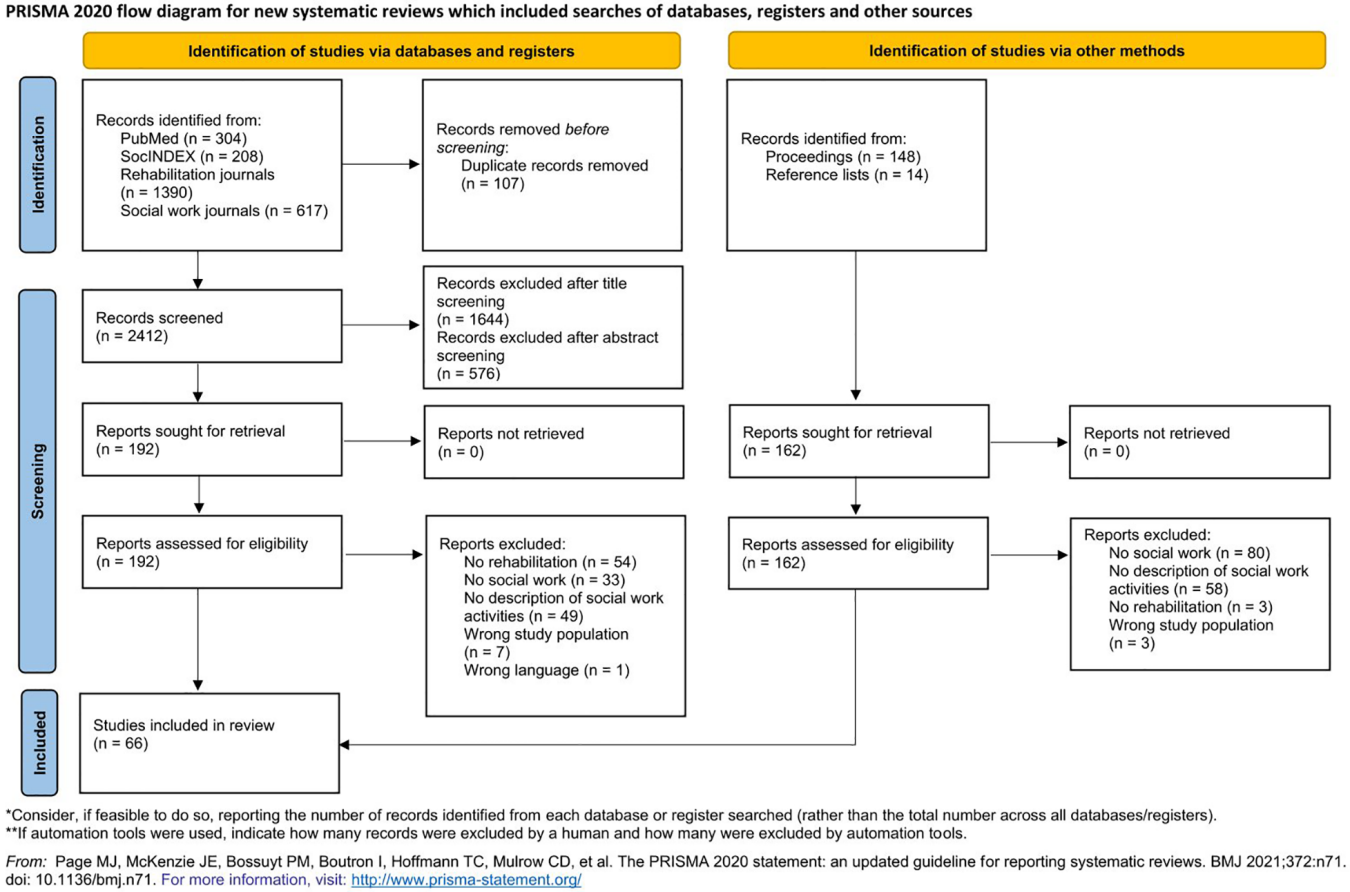
PRISMA flow-chart.

A total of 2,681 records could be identified by searching the databases, journals, proceedings and reference lists. After removing duplicates, 2,574 records remained for title- and abstract screening during which 2,220 records were excluded. Overall, the full-texts of 354 records were screened by the two authors. 66 sources met the predefined inclusion criteria of which 48 derived from the databases/journals search and 18 from the proceedings/reference lists search. Social workers were mentioned in additional 107 sources as members of the rehabilitation team (see Figure 1). However, due to no description of their activities, respective sources had to be excluded.

### Study characteristics

**Figure 2:**
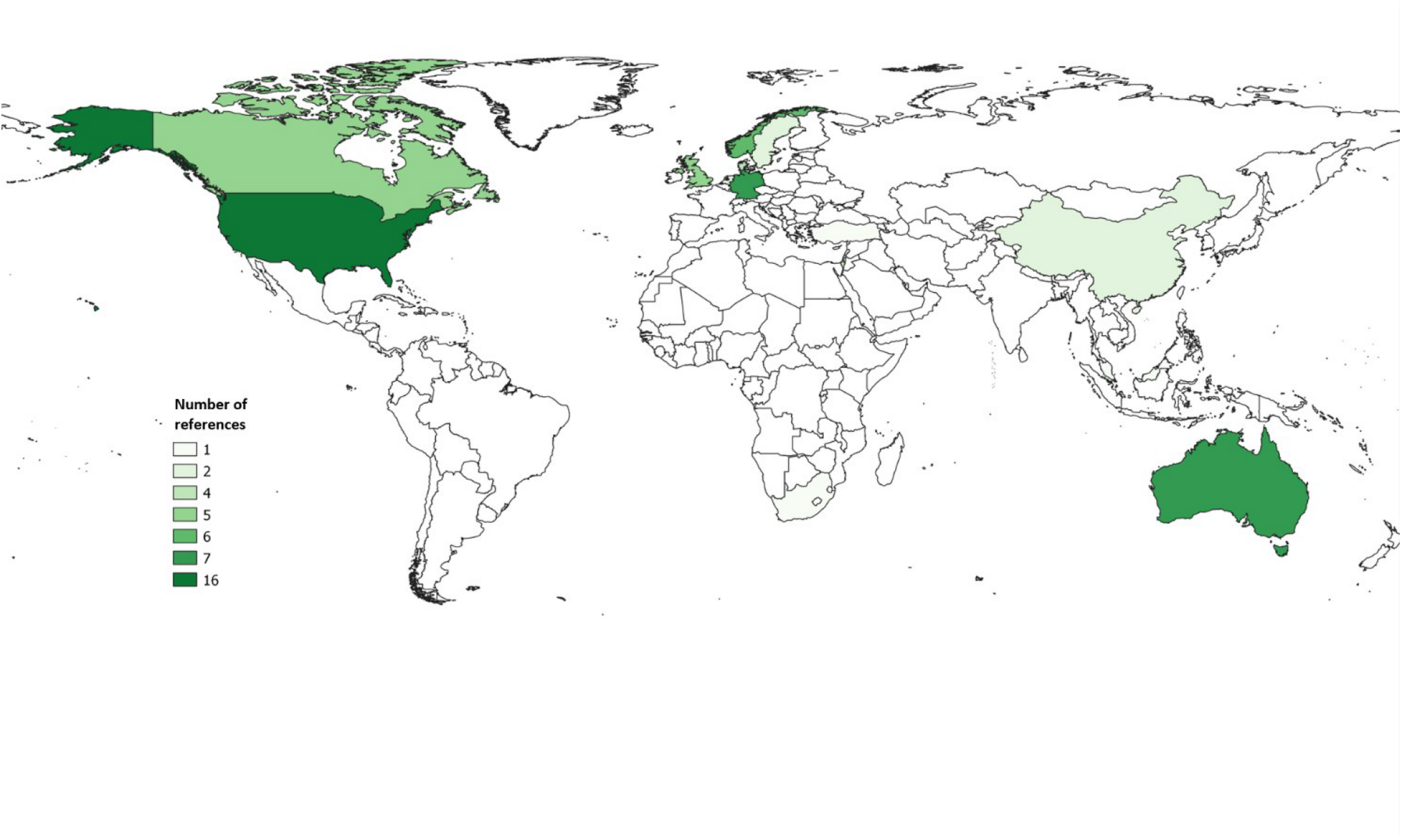
Countries from which the included references originate.

As shown in Figure 2, the identified literature references originate mainly from the USA (*n* = 16) (34–49), followed by Australia and Germany (both each *n* = 7) (12, 50–62). The remaining sources are mostly distributed between Canada (*n* = 5) (63–67) and European countries other than Germany (*n* = 20) (68–87), especially Scandinavian countries (*n* = 12) (68, 69, 72, 74, 75, 76, 77, 78, 81, 82, 83, 86). Few literature references come from Israel (*n* = 4) (88–91) and China (*n* = 2) (92, 93) and one from Malaysia (*n* = 1) (94), one from Turkey (*n* = 1) (87) and one from South Africa (*n* = 1) (95). Three sources refer to several countries (96–98) and are therefore not included in the map. For instance, Mantell et al. (98) conducted a literature review that included different studies from different locations or Zarshenas et al. (97) compared components of inpatient rehabilitation between Canadian and US rehabilitation facilities.

The majority of references refer to patients with neurological conditions (*n* = 23) (39, 40, 42, 45–47, 49, 50, 54–56, 60, 64, 70, 71, 73, 79, 80, 85, 91, 96–98). Among them, 17 references focus on patients with acquired brain injuries (ABI) (40, 42, 45, 47, 49, 50, 54–56, 70, 71, 73, 79, 85, 96–98) and three on stroke patients (46, 60, 91). With a noticeable gap compared to the quantity of sources covering neurological disorders, oncological (*n* = 11) (57, 58, 63, 65–67, 69, 72, 81, 83, 84), mental (*n* = 8) (37, 38, 41, 88, 89, 92, 93, 95), musculoskeletal (*n* = 4) (62, 77, 78, 87) and cardiological (*n* = 3) (43, 61, 90) disorders follow.

In terms of the setting rehabilitation measures take place, the largest share was provided in a clinical inpatient setting (*n* = 25) (12, 39, 43–45, 47, 48, 53–55, 57–59, 61, 69, 72, 74, 75, 81, 84, 86–88, 94, 97). 10 sources describe a community setting (40, 50, 51, 56, 70, 71, 85, 89, 92, 95), while 5 refer to an outpatient setting (37, 38, 52, 80, 91). 3 sources note a home-based (34, 35, 46), and 2 a digital/telephone setting (62, 63). 9 nine literature references focus on vocational rehabilitation (41, 68, 76–79, 82, 83, 93).

As far as the duration of rehabilitation measures is concerned, differences can be observed. The duration ranges from one week to up to two years. Country-specific patterns can only be identified for Germany, where inpatient rehabilitation measures usually last three to five weeks, depending on the indication. A total of 26 references indicate a duration of no more than twelve weeks (12, 39, 40, 44, 46, 52, 55, 57–59, 61, 64–69, 72, 80, 81, 84, 86, 87, 91, 95, 97). In contrast, in 7 sources it is stated that rehabilitation measures last from several months up to two years (37, 38, 62, 63, 88, 90, 93), while 4 sources report that the duration is individualized for each rehabilitant (41, 50, 70, 79). For instance, participants of a rehabilitative employment program attended the program until they returned to work (41). The remaining sources provide no information on the duration of the measures.

Since there were no restrictions regarding study designs, a broad spectrum could be found. These encompassed, e.g., 15 RCTs (37, 38, 40, 46, 52, 58, 61, 62, 68, 77, 80, 81, 84, 87, 90), 6 NRSI (42, 49, 57, 66, 79, 83), 7 studies with a single arm pre-/post design (64, 65, 67, 69, 72, 91, 93), 13 observational studies (34, 39, 44, 45, 54–56, 59, 60, 70, 74, 75, 97), 6 qualitative studies (36, 76, 82, 86, 92, 95) and 5 case reports (47, 73, 85, 94, 96). A more detailed overview of study characteristics is available in the Supplementary Material (Tables S4 and S5).

### Designation of social workers

All identified records provide a description of social workers working in rehabilitation and the tasks they carry out. Yet, the professionals are not always merely labeled as social workers. On the contrary, a range of designations and additional qualifications can be found in the sources. Besides the mere designation as “social workers”, professionals were linked to the field they are working in. For instance, they are called clinical or clinic-based social workers (34, 40, 43, 61, 90), rehabilitation social workers (89, 96), health social workers (74) or medical social workers (84). Additionally, some sources specify qualification-related backgrounds of the professionals. Examples are masters-level practitioners (36, 37, 42), licensed social workers (34, 40, 43) or the indication of experience in trauma intervention (41) or in treating eating disorders (88). Moreover, further specifications of social workers were denoted. Rosario et al. studied the effectiveness of a program for patients with TBI led by a so-called patient navigator who was a trained social worker (42). Other specifications included rehabilitation counselors (36), sexual health coaches (63), social service (87) or case mangers (39, 46, 48, 50, 70, 71, 77–79, 89, 90, 93, 97). The latter was the most common specification with 13 references directly referring to social workers as case managers or to case managers with a background in social work.

### Social work practice

A majority of the identified activities that social workers perform are case related. Topics that may occur in these case encounters are listed in Table 1. Financial/social security accounts for a large portion of these topics, with 21 publications addressing this topic as one that falls under the purview of social workers (34, 36, 39, 41–43, 45, 49, 54–56, 58, 60, 61, 75, 80, 85, 93–96). Activities arising from this issue may include assistance in applying for grants or social benefits. Other activities refer to counseling regarding financial planning or the mere education on social law-related topics.

**Table 1:** Social work topics.

Topics	Subtopics
Financial/social security	• Income generating activities • Financial planning/money management • Socio-legal topics (e.g., social benefits) • Insurance issues
Work-related issues	• RTW (including barriers and facilitators for RTW) • Acquiring, keeping, terminating a job • Professional biography • Current working situation
Disease-specific content	• Knowledge about the disease • Coping with the disease • Health promotion • Medical care
Social environment	• Social support • Social situations • Interpersonal relationships • Involvement of family members • Communication skills
Emotions/problems/conflicts	• Management of emotions/stress • Reflection of (problematic) situations • Problem solving/conflict resolution • Emotional support
Self-awareness/self-care	• Identity • Strategies for self-care • Self-advocacy • Individual resources and challenges • Spirituality

Aside from financial/social security, work-related issues are also a common area of responsibility for social workers. For instance, the professionals may be involved in the RTW process by developing, coordinating and tailoring RTW plans together with patients, the rehabilitation team and/or employers. Accordingly, the exchange with and involvement of the latter may also be a task of social workers in rehabilitation. Especially, if RTW is formulated as a rehabilitation goal, respective plans have to be reconciled with the employer. Social workers can take on this task. Another possibility of engaging the employer, are consultations and education concerning the RTW of employees with health conditions. In total, 21 references mentioned work-related issues as a social work responsibility making them a relevant scope of duties (12, 36, 41, 56–59, 61, 62, 68, 75–78, 80, 81, 83, 89, 90, 92, 93).

Since rehabilitation takes place in a health-related context (26), its target group has one or more health conditions. Disease-specific content is thus a social work concern in rehabilitation, as shown by 15 sources (37–41, 54, 60, 61, 77, 78, 80, 88, 90, 95, 96). In some references, social workers educated patients about their disease. For instance, social workers facilitating an enriched supportive therapy (EST) group for patients with schizophrenia, provided psychoeducation about the disease, including informing patients about symptoms and their triggers (37, 38). Other activities encompass addressing health-promoting behaviors and education about coping strategies for the disease. Precisely, in one reference it is stated that the participants of a community-based psychosocial rehabilitation program recognized the role of the social workers in teaching and sharing information about coping with schizophrenia (95).

Further tasks stem from the patients’ social environment. As part of the assessment, social workers may address the patients’ social network. For example, a social work intervention in cardiac rehabilitation addressed social support and in this context one's own awareness of the social network and the activation of available social support resources (61). In order to improve social competencies and interpersonal relations, social workers also provide communication training and conduct individual and group sessions regarding social expectations and norms (40, 58, 61, 76, 88). In addition, family/caregiver involvement can also be part of social work practice. This takes the form of counseling and educational sessions offered not only to the patient but also to his/her family/caregiver. Some sources even introduce interventions targeting the patient's social environment. For instance, a Short Stay Family Training program addressed patients with terminal illnesses as well as their family/caregivers (44). Altogether, 26 references addressed the social environment of the rehabilitants (34, 37, 38, 40–42, 44–46, 49, 50, 54, 55, 61, 64, 71, 74–76, 80, 87–90, 96, 98).

The already frequently mentioned consultation and education by social workers also refers to the topics of emotions, problems and conflicts that can be found in 17 sources (36–38, 41, 44, 45, 51, 55, 56, 58, 61, 80, 84, 86, 87, 93, 95). Social workers in rehabilitation may conduct educational sessions on anger and stress management. They address conflicts in the area of the family as well as in working life and provide crisis intervention.

Another area that is covered by social work practice in rehabilitation is the patient's self, or more specifically, their self-awareness, self-efficacy and self-care. 10 references have focused on this topic (36–38, 40, 41, 61, 74, 87, 92, 95). In this regard, social workers address strategies for self-care and self-organization, taking responsibility for one's own life, spirituality and challenging patients to reflect on their own strengths and challenges.

In summary, some of the presented social work activities cross the subject areas listed in Table 1. Of particular note are activities like counseling and education. However, activities related to the conduction of assessments are also cross-thematic. Social workers are involved in the assessment conducted in the rehabilitation setting. More precisely, some of the identified records mention biopsychosocial (46) respectively psychosocial assessments (34, 51, 54, 55, 97) as well as assessments of risks and needs (51, 73). Since social workers are part of an interprofessional team, they are not the only ones conducting assessments of patients. Rather, a profession-specific assessment can be observed. Assessed areas by social work may include the vocational background of patients, their readiness for RTW, perceived difficulties regarding RTW and their work situation including tasks. However, patient's social network, home environment, service needs and current health status as well as perceptions of their disease and its impact on their lives and family may also be assessed by social workers.

Based on the assessments, social workers can also be involved in developing rehabilitation plans and setting rehabilitation goals together with patients and the rehabilitation team. For instance, goals can be discussed, reviewed and modified with the patient and sometimes even together with the family/caregiver (12, 40, 44, 46, 62, 71, 80, 85, 88, 95).

For further work with patients, some of the identified references emphasize the need for social workers to build a working alliance with patients as well as their family/caregiver and other members of the rehabilitation team. Building this kind of working alliance can have several facets, such as advocating for the patient and his/her rights, creating a safe environment and gaining trust. However, engaging communication is also highlighted. This encompasses, for example, a supportive and active style of listening, as well as validating emotions (36, 41). The latter can also be counted among the therapeutic activities that social workers may perform. For instance, social workers received a four-day training in the use of elements of Acceptance and Commitment Therapy that was led by a psychologist (68). In an RCT, schizophrenia patients were randomly assigned to a Cognitive Enhancement Therapy (CET) group and an EST group that served as control group. The effects of CET on employment outcomes were examined. Both groups, CET and EST, were facilitated by masters-level social workers who were trained and supervised by the developers of CET and EST. As part of CET, social workers have led social-cognitive groups. These groups pursued a rather educational approach and covered themes such as perspective-taking or emotion management (37, 38). In a cardiac pulmonary rehabilitation center, social workers provided mental health services. The professionals were at least masters-level students and were supervised by a professional who was a masters-level practitioner in social work and had a Ph.D. in Clinical Psychology as well as training in cognitive behavioral therapy and motivational interviewing (43). In addition, another source states that a clinical social worker who served as a case manager in a case management rehabilitation program provided psychotherapeutic treatment when needed (90).

As already mentioned with regard to the designations of social workers and also stated in the example above, the role of a case manager can be fulfilled by social workers. For instance, one activity that is part of case management in the rehabilitation context is coordination of services and, in particular, coordination and communication between patients, their families/caregivers and providers. Other activities that may occur but are not solely specific to case management include conducting assessments, counseling, education, making referrals to other providers as needed and discharge planning. Engaging community resources can also be a task of the social worker/case manager. For example, social workers may activate community-based service providers who offer and ensure post-rehabilitation care. However, social workers can also be directly involved in aftercare activities themselves. Vogel et al. (2017) examined a telephone-based follow-up intervention delivered by social workers. Social workers called the rehabilitants at 2-month intervals for one year. The telephone conversations focused on the current status of vocational integration, the realization of pre-defined goals and the mutual development of further strategies to achieve these goals (62).

As mentioned earlier, social workers collaborate not only with rehabilitants, but also with the rehabilitation team. The collaboration can take various forms. One common form is represented by team meetings. Team meetings can be organized as interdisciplinary case conferences where topics such as goal setting, discharge planning and medical issues can be discussed (39, 53, 78, 97). Also, assessed information about patients can be exchanged and rehabilitation plans can be jointly developed based on this information (51, 74, 78, 96). Overall, these meetings can be a mean of consultation and support. Other forms of cooperation include joint meetings with rehabilitants and their families, which in some cases are organized by social workers themselves (55). Some of the identified records even displayed the mutual conduction of interventions (41, 49, 57, 68, 87, 88). For instance, an occupational therapist co-led trauma focused group therapy sessions together with a social worker. There were no differences in the roles of the two professionals (41). Another example of a co-led group is a so-called back-to-work group led by a psychologist and social worker. Participants discussed RTW and related emotions with the psychologist and social worker. If needed, the social worker additionally offered job-related counseling and discussion of gradual reintegration (57). A jointly run group was also in the focus of Akgül Gök et al. (87). The authors investigated the effectiveness of an empowerment intervention program that was co-led by social workers and nurses.

All the activities presented so far have been more or less case-related. The interaction setting has already become visible at one point or another. A rough subdivision can be made into group and individual settings. Communication respectively interaction took place *via*:

• personal contact in the rehabilitation facility,
• home visits,
• telephone/video calls,
• e-mail,
• applications.

Some of the sources indicated that digital forms of communication with the rehabilitants or the rehabilitation team were necessary due to the COVID-19 pandemic (51, 53).

Eventually, some non-case-related social work activities in rehabilitation could be identified. Shah et al. (2019) discussed the role of social work in cardiac rehabilitation settings for older adults. The authors highlighted the mental health needs of rehabilitants and how social workers can improve care. In this regard, they note that social workers can assist with building networks for cardiac rehabilitation facilities, particularly in relation to mental health care providers. Additionally, the authors presented a project where social workers trained cardiac rehabilitation staff. They helped to implement screening tools for mental health and guided staff in the utilization of these tools and the recognition of mental health issues (43).

### Social work outcomes

**Figure 3:**
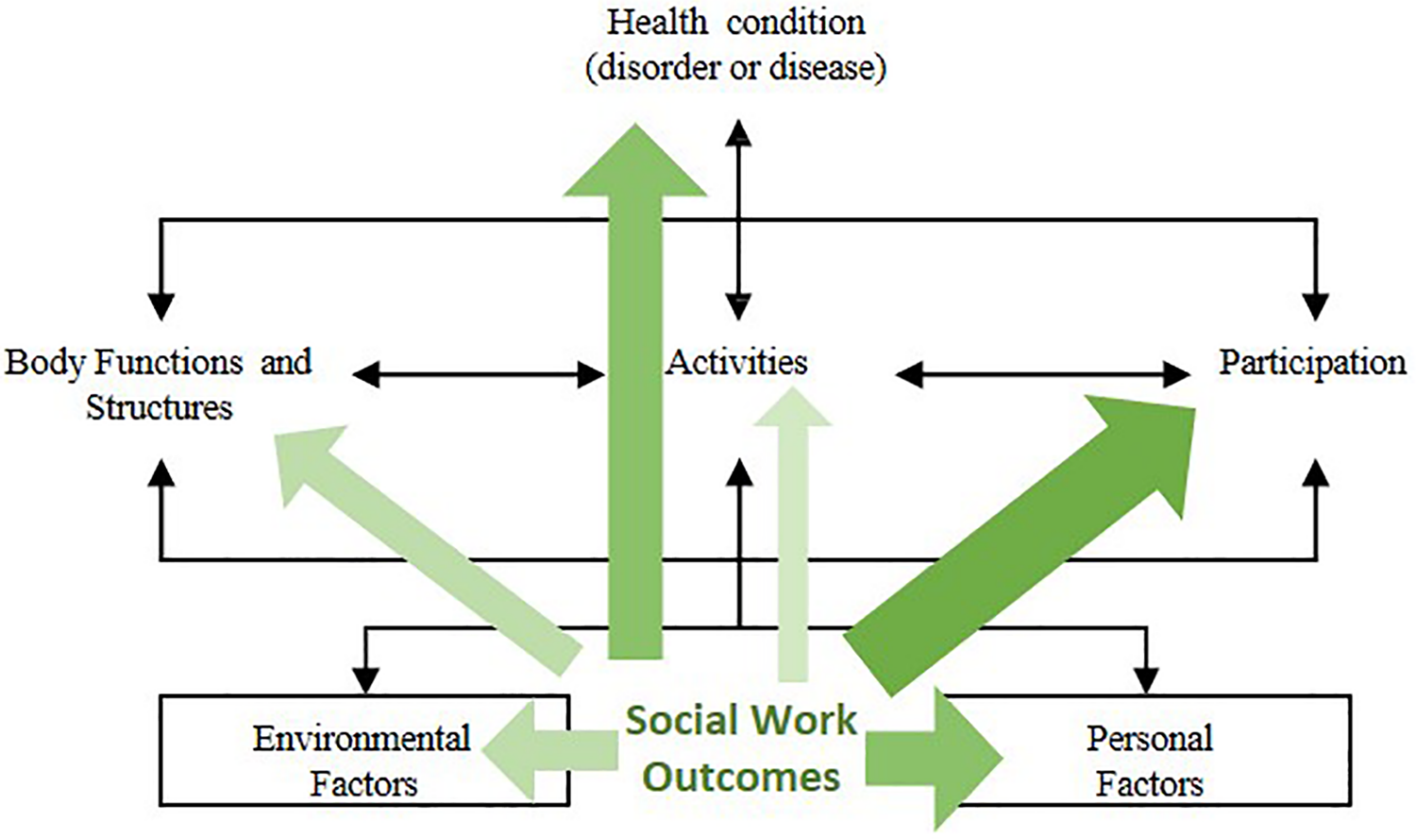
Identified outcomes linked to components of the ICF (adapted illustration of the authors, 33).

Social work outcomes were only extracted from references that focused on interventions solely carried out by social work professionals. In total, outcomes could be extracted from 13 articles (37, 38, 40, 42, 46, 54, 59, 61, 62, 66, 83, 90, 91). The outcomes thus identified were linked to the components of the ICF. As can be seen in Figure 3, outcomes could be linked to all components except from *Body Structures*. The volume and the color of the arrows indicates how often outcomes linked to a specific component were reported. The larger the volume and the darker the color, the more often outcomes were reported.

An emphasis on the component *Participation* can be observed. 9 out of 13 articles reported outcomes in this area (37, 40, 42, 46, 59, 61, 62, 83, 90). Predominantly, outcomes linked to this component fall under vocational participation. Authors of respective studies reported e.g., RTW or employment characteristics in general. Besides outcomes related to vocational participation, social functioning and social role participation are reported outcomes that fall under this component. Following *Participation*, the most frequently reported outcomes could be linked to the areas of *Personal Factors* (40, 61, 62, 66, 83, 91) and *Health Condition* (42, 46, 59, 62, 66, 90). 6 studies each, reported corresponding outcomes. Outcomes related to *Personal Factors* included e.g., the subjective prognosis of employability, readiness for RTW, comorbidities or self-advocacy. The latter includes the perceptions of one's own needs and the ability to address these as well as to cope with demands in life and the perceived success in doing so. For instance, Hawley et al. evaluated the efficacy of an intervention to enhance self-advocacy for people with TBI using the Self-Advocacy Scale (40). Outcomes that were grouped under *Health Condition* comprise outcomes referring e.g., to the overall health, the psychological state or the occurrence of rehospitalizations.

Less frequently, outcomes linked to *Body Functions* (37, 38, 42), *Environmental Factors* (46, 62, 90) and *Activities* (42, 59) could be identified. In the case of *Body Functions*, 2 references, referring to the same RCT, report that cognitive assessments were conducted (37, 38), e.g., assessments of attention, memory and executive functioning. In addition, Rosario et al. assessed the neuroendocrine status of participants with TBI of a patient navigation intervention (42). Further 3 studies reported outcomes in the area of *Environmental Factors*. Identified outcomes were the utilization of social benefits after a social work intervention (62), the occurrence of a meeting with an occupational physician (90), the perceived availability of helpful information/advice as well as the sense of being looked after and cherished as a person (46). Only 2 studies reported outcomes that could be linked to *Activities*. These include Activities of Daily Living and Falls (42, 59).

Beyond that, outcomes could be identified that could not be linked to the components of the ICF. This includes life satisfaction respectively well-being (40, 46, 66), health-related quality of life (61, 91), patient satisfaction (54) and caregiver burden (42). The latter study, which reported caregiver burden, is 1 out of 2 studies that refers to caregiver outcomes. In addition to caregiver burden, social functioning, well-being and physical as well as mental health of caregivers could be identified as outcomes of caregivers (46). A detailed overview of activities and outcomes by reference can be found in the Supplementary Material (Tables S6 and S7).

## Discussion

This scoping review, which identified 66 studies published in the last decade, demonstrates the role of social work in the interprofessional rehabilitation team. In addition to the 66 identified references, 107 references mentioned social work as part of the interprofessional rehabilitation team, but did not further characterize the activities performed and were therefore excluded. Taken together, the findings of the review lead to the conclusion that social work is an integral and vital part of the interprofessional rehabilitation team. This applies to a variety of world regions, albeit with a focus on high-income countries. The underlying two research questions that were guiding for performing the scoping review could be answered. We were able to identify and map social work activities and reported outcomes. A variation was observed in terms of the rehabilitation settings in which social work practice occurred. Settings included inpatient medical rehabilitation, community-based rehabilitation or vocational rehabilitation. There was also a wide range of medical indications, although the majority of articles related to patients with neurological conditions. The data showed further variations in the training of the professionals and the related assignment with therapeutic tasks. Despite these variations, some core social work activities and topics were detected. These were, inter alias, the acquiring of financial/social security, addressing the social environment and work-related issues, counseling and discharge planning. When considering the ICF framework, the described outcomes are distributed across almost all components with an emphasis on *Participation*.

This paper adds to the body of knowledge in rehabilitation science with research evidence from a profession that has received little attention in the past. This is in accordance to the Rehabilitation 2030 initiative (99), which aims at developing a strong multidisciplinary rehabilitation workforce especially through research activities. At the same time, the under-representation of included references from low-income countries contradicts the initiative and underlines a prominent problem in global research. Other international reviews as well as specialized social work reviews also reported an underrepresentation of low-income countries (100, 101). The contextual nature of social work activities (102) limits the scope of the results to high and middle-income countries. In particular, the international comparison leads to problems in the transferability of the results in social work education. The licensing of social workers in the USA often leads to a therapeutic approach (e.g., 38) that is intended for other professions in many other countries. This is also accompanied by special and other activities and outcomes of these. The core activities described here underline the results of a conceptual study, that identified similar activities and outcomes as a central part of a program theory for social work in medical rehabilitation (17). Additionally, the activities identified in this scoping review are related to so-called core activities, such as client advocacy, counseling, information, and empowerment that were found in a study by Sommerfeld et al. that aimed to explore the mechanisms of action of social work in a health care context (103). There are also similarities with the activities and outcomes of other health-related social work interventions (21, 104). Fugl-Meyer (105) as well as Knoop & Meyer (59) have linked social work to the biopsychosocial model of the ICF. The results of this scoping review indicate that social work can have an impact on several components of the ICF. The emphasis on outcomes in the area of *Participation* was to be expected, as social work addresses people who are impaired in their participation (4). Nonetheless, *Environmental Factors* were not expected to play a such a minor role. This is particularly surprising given the historic significance of the Person-in-Environment approach in social work as a central framework for the profession (106). Complementary, 27 references addressed the social environment and further 21 focused on the financial/social security of patients which ties in with findings of Knoop et al. (17). This suggests that future studies, especially if the content of the intervention addresses contextual factors, should report more outcomes related to *Environmental Factors*. Furthermore, a yet to be developed outcome model for social work in rehabilitation could map potentially relevant outcomes and their interrelation, enabling researchers to select appropriate outcomes.

The search strategy of the scoping review favored some indication-specific journals. This may have contributed to the overrepresentation of neurological conditions in the sample. This may also apply to oncological rehabilitation, although it was not reflected in the findings. The use of the definition of rehabilitation by Negrini et al. (27) was helpful in conducting the review, although we cannot rule out that all applicable studies were found, as the studies often did not specify the study setting properly. We cannot be sure, that the mention of the term *social work* is an indication of the actual involvement of social workers. However, our approach is comparable to that of other reviews on related topics (21). In contrast to other reviews, we included like Knoop et al. (12) a large proportion of the references via direct access to the registries of the included journals. For the German context, this is due to the lack of sufficient infrastructures in social work research (107). It is possible that the review presented here links two areas that have not yet made enough reference to each other. The deductive approach when analyzing the reported outcomes was not fully sufficient. For instance, we had difficulties in assigning rehospitalizations to the ICF framework. We considered this outcome as an indicator for the patients’ health status and assigned it respectively to *Health Condition*. Corresponding to the definition of the ICF categories, one could have assigned it to *Environmental Factors* as well (see e5800 in the ICF, 33). An alternative approach would have been the use of the capability approach framework (108). Furthermore, the number of references that indicate certain activities or reported outcomes only provide a general direction and should be interpreted cautiously, since the data was not “cleansed” in the sense that multiple sources referring to a single study were only counted as one.

This scoping review does not make any assumptions about the efficacy or effectiveness of social work interventions. Especially for the neurological rehabilitation, we believe there is enough potential to perform a meta-analysis of the overall effectiveness. Other reviews reported positive effects and mixed effects (12, 104, 109). Experimental research in social work is often considered impossible (110). This review shows the possibility and potential of intervention and evaluation research in social work. We must note that further studies on the effectiveness of social work in rehabilitation should be conducted. The outcomes collected here should guide further research. Social work interventions appear as complex interventions in the light of the data presented here. Accordingly, the theoretical background as well as the development and plausibility of mechanisms of action are of particular importance (6, 111). With its conceptual analysis approach, this review offers a special contribution to the field of social work in rehabilitation by detecting core activities, recurring topics and possible outcomes. Given the evidence suggesting a potential lack of understanding regarding social work's potential roles and tasks in an interprofessional context (19, 20), this review may also serve to provide rehabilitation practitioners insight into the potential roles social workers can undertake in the rehabilitation process and their corresponding competencies. Consequently, it could facilitate interprofessional collaboration by promoting role clarity.
