# Supplementary material for: Social work practice and outcomes in rehabilitation: a scoping review
Source: Front Rehabil Sci. 2024 Nov 18;5:1348294. doi: 10.3389/fresc.2024.1348294 (PMC11609207; doi:10.3389/fresc.2024.1348294)
Supplement: Supplementary file 1 [file Table1.docx]

Supplementary Material

# Supplementary Tables

Table 1 Search strategies (PubMed and SocINDEX)

| **Search strategies** | |
| --- | --- |
| PubMed | SocINDEX |
| (("Social Work" [Mesh]) OR ("Social Work" [Title/Abstract])) AND ("Physical and Rehabilitation Medicine" [Mesh] OR "Rehabilitation" [Mesh]) AND (English [lang] OR German [lang]) AND ("2010/01/01" [PDAT]: "3000/12/31" [PDAT]) | (DE "SOCIAL workers" OR “social work”) AND (DE "REHABILITATION" OR DE “VOCATIONAL rehabilitation”) |

Table 2 List of searched journals

| **list of searched journals** | |
| --- | --- |
| rehabilitation journals (search term: “social work”) | social work journals (search term: rehabilitation) |
| 1. Disability & Rehabilitation 2. Topics in Stroke Rehabilitation 3. Disability & Society 4. Journal of Occupational Rehabilitation 5. Supportive Care in Cancer 6. Clinical Rehabilitation 7. Psychiatric Rehabilitation Journal 8. BMC Sports Science, Medicine and Rehabilitation 9. American Journal of Physical Medicine & Rehabilitation 10. PM & R 11. Rehabilitation Counseling Bulletin 12. Annals of Physical and Rehabilitation Medicine 13. Archives of Physical Medicine and Rehabilitation 14. Disability and Health 15. Journal of Rehabilitation Medicine 16. Neurorehabilitation 17. Psycho-Oncology (search terms: “Social Work” AND Rehabilitation) 18. Frontiers in Rehabilitation Science | 1. Health and Social Care in the community 2. Social Service Review 3. Journal of the Society for Social Work and Research 4. Journal of Social Work 5. Qualitative Social Work 6. International Social Work 7. Research on Social Work Practice 8. British Journal of Social Work 9. Social Work Research 10. Social Work Research and Abstracts 11. Health & Social Work 12. Social Work 13. Journal of Social Service Research 14. Journal of Social Work Education 15. Journal of Gerontological Social Work 16. Social Work in Health Care 17. Journal of Social Work Practice 18. Journal of Community Practice 19. Social Work in Public Health 20. Journal of Evidence Based Social Work 21. Human Service Organizations 22. Australian Social Work 23. European Journal of Social Work 24. Social Work in Mental Health 25. Social Work and Social Sciences Review 26. Social Work and Society 27. Advances in Social Work 28. Clinical Social Work 29. Journal of Social Policy 30. Social Policy and Society 31. Journal of Sociology & Social Welfare 32. Journal of Social Work in Disability and Rehabilitation |

Table 3 List of searched proceedings

| **list of searched proceedings** |
| --- |
| 1. Annual Meeting of the Association of Academic Physiatrists 2. British Society of Rehabilitation Medicine: Annual Meeting 3. International Society of Physical and Rehabilitation Medicine: World Congress 4. Multinational Association of Supportive Care in Cancer 5. World Congress on Brain Injury |

Table 4 Characteristics of included literature references (solely social work involvement)

| **solely social work involvement** | | | | | |
| --- | --- | --- | --- | --- | --- |
| **authors and year** | **country** | **indication** | **rehabilitation setting** | **duration** | **study design** |
| Brooke-Sumner et al. (2017) | South Africa | mental disorders | community setting | 4 weeks | qualitative study |
| Eack et al. (2011) | USA | mental disorders | outpatient setting | 2 years | RCT |
| Eack et al. (2016) | USA | mental disorders | outpatient setting | 2 years | RCT |
| Edelmaier (2021) | Australia | / | community setting | / | report |
| Hammond et al. (2011) | USA | neurology | inpatient setting | 10 hours, 72.8 minutes per week | observational study |
| Hawley et al. (2022) | USA | neurology | community setting | 10 weeks | RCT |
| Holloway & Fyson (2016) | UK | neurology | several settings | / | case report |
| Holloway & Tyrrell (2016) | several countries | neurology | several settings | / | case report |
| Knoop et al. (2019) | Germany | several indications | inpatient setting | 3 – 5 weeks | review |
| Knoop & Meyer (2020) | Germany | several indications | inpatient setting | 3 weeks | observational study |
| Larsson et al. (2019) | Sweden | chronic pain | inpatient setting | / | observational study |
| Lehnerer et al. (2019) | Germany | neurology | several settings | / | observational study |
| Lutsky et al. (2013) | Israel | neurology | outpatient setting | once a week for 1 hour | single arm pre-/post design |
| Mantell et al. (2012) | several countries | neurology | / | / | review |
| Martin-MacKay et al. (2012) | Canada | oncology | / | 8 weeks | NRSI |
| Michelsen et al. (2020) | Norway | cognitive disabilities | vocational rehabilitation | / | qualitative study |
| Olivieri (2020) | Australia | / | inpatient setting | / | report |
| Roberts et al. (2012) | Australia | neurology | inpatient setting | / | observational study |
| Rosario et al. (2017) | USA | neurology | several settings | / | NRSI |
| Salzwedel et al. (2019) | Germany | cardiology | inpatient setting | 3 weeks | RCT |
| Shah et al. (2019) | USA | cardiology | inpatient setting | / | report |
| Simpson et al. (2016) | Australia | neurology | inpatient setting | 12 weeks | observational study |
| Solberg (2011) | Norway | several indications | vocational rehabilitation | / | qualitative study |
| Stapelfeldt et al. (2021) | Denmark | oncology | vocational rehabilitation | / | NRSI |
| Vogel et al. (2017) | Germany | orthopedics | telephone-based aftercare | 1 year | RCT |
| Vungkhanching et al. (2016) | USA | neurology | inpatient setting | / | observational study |
| Wiseman (2011) | UK | neurology | community setting | / | case report |
| Woodward et al. (2021) | USA | neurology | home-based | 60 days | RCT |
| Yerushalmi (2016) | Israel | mental disorders | community setting | / | report |
| Zack et al. (2022) | Israel | cardiology | several settings | up to 2 years | RCT |

Table 5 Characteristic of included literature references (social work as part of the intervention)

| **social work as part of the intervention/rehabilitation team** | | | | | |
| --- | --- | --- | --- | --- | --- |
| **authors and year** | **country** | **indication** | **rehabilitation setting** | **duration** | **study design** |
| Aasdahl et al. (2021) | Norway | several indications | vocational rehabilitation | 6-7 weeks | RCT |
| Akgül Gök et al. (2023) | Turkey | orthopedics | inpatient setting | 2 weeks | RCT |
| Bertheussen et al. (2012) | Norway | oncology | inpatient setting | 3 weeks and 1 week follow-up | single arm pre-/post design |
| Böttcher et al. (2013) | Germany | oncology | inpatient setting | 3 weeks | NRSI |
| Butera-Prinzi et al. (2010) | Australia | neurology | community setting | Individual | mixed-methods design |
| Campbell et al. (2015) | USA | caregiver burden | home-based | / | observational study |
| Campbell et al. (2014) | USA | caregiver burden | home-based | / | report |
| Chasen et al. (2011) | Canada | oncology | / | 8 weeks | single arm pre-/post design |
| Chasen et al. (2015) | Canada | oncology | / | 8 weeks | single arm pre-/post design |
| Chin et al. (2014) | USA | neurology | inpatient setting | / | case report |
| Clark-Wilson (2016) | UK | neurology | community setting | individual | observational study |
| Clark-Wilson & Holloway (2015) | UK | neurology | community setting | / | report |
| Dispenza et al. (2016) | USA | chronic illness(es) and/or disabilities | several settings | / | qualitative study |
| Fauser et al. (2019) | Germany | inpatient setting | 3 weeks | 3 weeks | RCT |
| Foster & Rosenberg (2018) | USA | inpatient setting | / | / | quality improvement project |
| Girdler et al. (2010) | Australia | outpatient setting | 8 weeks | 8 weeks | RCT |
| Hauken et al. (2015) | Norway | oncology | inpatient setting | 3 weeks and 1 week follow-up | single arm pre-/post design |
| Jokel (2016) | Canada | neurology | / | ten 2-hour sessions (weekly) | single arm pre-/post design |
| Latzer (2019) | Israel | mental disorders | inpatient setting | up to 1 ½ year + 1 year follow-up | report |
| Li & Ma (2021) | China | mental disorders | community setting | / | qualitative study |
| Matthew et al. (2022) | Canada | oncology | digital | 1 year | feasibility study |
| Mengshoel & Skarbø (2017) | Norway | rheumatology | inpatient setting | / | observational study |
| Pedersen et al. (2017) | Denmark | orthopedics | vocational rehabilitation | / | study protocol |
| Pedersen et al. (2022) | Denmark | orthopedics | vocational rehabilitation | / | RCT |
| Precin (2011) | USA | mental disorders | vocational rehabilitation | 90 minutes once a week until RTW | report |
| Radford et al. (2013) | UK | neurology | vocational rehabilitation | individual | NRSI |
| Rietberg et al. (2014) | Netherlands | neurology | outpatient setting | < 12 weeks | RCT |
| Rosario et al. (2013) | USA | neurology | / | / | NRSI |
| Rottmann et al. (2012) | Denmark | oncology | inpatient setting | 1 week | RCT |
| Smith et al. (2020) | USA | oncology | inpatient setting | 1 week | observational study |
| Stigt et al. (2013) | Netherlands | oncology | inpatient setting | 12 weeks | RCT |
| Ta’eed et al. (2015) | Australia | neurology | community setting | / | observational study |
| Young et al. (2019) | China | mental disorders | vocational rehabilitation | several months | single arm pre-/post design |
| Yuliawiratman & Suhaimi (2015) | Malaysia | obesity | inpatient setting | / | case report |
| Zakrisson et al. (2013) | Sweden | pulmonology | inpatient setting | 6 weeks | qualitative study |
| Zarshenas et al. (2019) | several countries | neurology | inpatient setting | Canada: Ø 40 days | observational study |
|  |  |  |  | USA: Ø 14 days |  |

Table 6 Social work activities and outcomes

| **solely social work involvement** | | |
| --- | --- | --- |
| **authors and year** | **social work activities/*topics*** | **outcome domains** |
| Brooke-Sumner et al. (2017) | - facilitating groups - referral to other health care providers - goal setting - *financial/social security* - *disease-specific content* - *emotions, problems and conflicts* - *self-awareness/self-care* | / |
| Eack et al. (2011) | - therapeutic activities (facilitation of CET groups and EST via individual therapy sessions) - *disease-specific content* - *social environment* - *emotions, problems and conflicts* - *self-awareness/self-care* | - Body Functions - Participation |
| Eack et al. (2016) | - therapeutic activities (facilitation of CET groups and EST via individual therapy sessions) - *disease-specific content* - *social environment* - *emotions, problems and conflicts* - *self-awareness/self-care* | - Body Functions |
| Edelmaier (2021) | - conducting home visits and telephone appointments - conducting assessments - collaboration with the rehabilitation team - engaging community resources - *emotions, problems and conflicts* | / |
| Hammond et al. (2011) | - case management - discharge planning - engaging community resources - counseling - collaboration with the rehabilitation team - *financial/social security* - *disease-specific content* | / |
| Hawley et al. (2022) | - *facilitating groups* - *engaging community resources* - *goal setting* - *disease-specific content* - *social environment* - *self-awareness/self-care* | - Participation - Personal Factors - life satisfaction/well-being |
| Holloway & Fyson (2016) | - conducting assessments | / |
| Holloway & Tyrrell (2016) | - case management - collaboration with the rehabilitation team - conducting assessments - engaging community resources - *financial/social security* - *disease-specific content* - *social environment* | / |
| Knoop et al. (2019) | - conducting telephone appointments - counseling - goal setting - aftercare activities - *work-related issues* | / |
| Knoop & Meyer (2020) | - counseling - aftercare activities - *work-related issues* | - Activities - Participation - Health Condition |
| Larsson et al. (2019) | - conducting assessments - collaboration with the rehabilitation team - documentation - *social environment* - *self-awareness/self-care* | / |
| Lehnerer et al. (2019) | - engaging community resources - *financial/social security* - *disease-specific content* | / |
| Lutsky et al. (2013) | - facilitating groups | - Personal Factors - health-related quality of life |
| Mantell et al. (2012) | - facilitating groups - case management - counseling - education - *social environment* | / |
| Martin-MacKay et al. (2012) | - counseling - therapeutic activities | - Personal Factors - Health Condition - life satisfaction/well-being |
| Michelsen et al. (2020) | - communication via applications - *work-related issues* - *social environment* | / |
| Olivieri (2020) | - conducting telephone and video appointments - building a working alliance - collaboration with the rehabilitation team - referral to other providers | / |
| Roberts et al. (2012) | - conducting assessments - discharge planning - *financial/social security* - *disease-specific content* - *social environment* | - patient satisfaction |
| Rosario et al. (2017) | - conducting telephone appointments - coordinating of care - engaging community resources - collaboration with the rehabilitation team - *financial/social security* - *social environment* | - Body Functions - Activities - Participation - Health Condition - caregiver burden |
| Salzwedel et al. (2019) | - facilitating groups - counseling - *financial/social security* - *work-related issues* - *disease-specific content* - *social environment* - *emotions, problems, conflicts* - *self-awareness/self-care* | - Participation - Personal Factors - health-related quality of life |
| Shah et al. (2019) | - conducting assessments - therapeutic activities - referral to other providers - cross-case activities - *financial/social security* | / |
| Simpson et al. (2016) | - conducting assessments - counseling - education - case management - referral to other providers - discharge planning - building a working alliance - collaboration with the rehabilitation team - conducting home visits - conducting telephone appointments - *financial/social security* - *social environment* - *emotions, problems, conflicts* | / |
| Solberg (2011) | - counseling | / |
| Stapelfeldt et al. (2021) | - conducting assessments - therapeutic activities - collaboration with the rehabilitation team - referral to other providers - *work-related issues* | - Participation - Personal Factors |
| Vogel et al. (2017) | - conducting telephone appointments - counseling - goal setting - aftercare activities - *work-related issues* | - Participation - Environmental Factors - Personal Factors - Health Condition |
| Vungkhanching et al. (2016) | - facilitating groups - counseling - discharge planning - referral to other providers - building a working alliance - *financial/social security* - *social environment* - *emotions, problems, conflicts* | / |
| Wiseman (2011) | - case management - coordinating care - collaboration with the rehabilitation team - goal setting - *financial/social security* | / |
| Woodward et al. (2021) | - conducting home visits - case management - conducting assessments - goal setting - *social environment* | - Participation - Environmental Factors - Health Condition - life satisfaction/well-being |
| Yerushalmi (2016) | - case management - collaboration with the rehabilitation team - building a working alliance - *work-related issues* - *social environment* | / |
| Zack et al. (2022) | - case management - coordinating care - conducting assessments - referral to other providers - therapeutic activities - aftercare activities - *work-related issues* - *disease-specific content* - *social environment* | - Participation - Environmental Factors - Health Condition |

Table 7 Social work activities

| **social work as part of the intervention/rehabilitation team** | |  |
| --- | --- | --- |
| **authors and year** | **social work activities/*topics*** | |
| Aasdahl et al. (2021) | - collaboration with the rehabilitation team - documentation - *work-related issues* | |
| Akgül Gök et al. (2023) | - facilitating groups - education - collaboration with the rehabilitation team - building a working alliance - *social environment* - *emotions, problems and conflicts* - *self-awareness/self-care* | |
| Bertheussen et al. (2012) | - facilitating groups - education - consultation | |
| Böttcher et al. (2013) | - facilitating groups - collaboration with the rehabilitation team - referral to other providers - *work-related issues* | |
| Butera-Prinzi et al. (2010) | - *social environment* | |
| Campbell et al. (2015) | - conducting home visits - conducting assessments - referral to other providers - *financial/social security* - *social environment* | |
| Campbell et al. (2014) | - conducting home visits - conducting assessments | |
| Chasen et al. (2011) | - conducting assessments | |
| Chasen et al. (2015) | - conducting assessments | |
| Chin et al. (2014) | - discharge planning | |
| Clark-Wilson (2016) | - case management - coordinating care - counseling | |
| Clark-Wilson & Holloway (2015) | - case management - coordinating care - conducting assessments - goal setting - building a working alliance - *social environment* | |
| Dispenza et al. (2016) | - conducting assessments - counseling - building a working alliance - financial/social security - work-related issues - emotions, problems and conflicts - *self-awareness/self-care* | |
| Fauser et al. (2019) | - counseling - *financial/social security* - *work-related issues* - *emotions, problems and conflicts* | |
| Foster & Rosenberg (2018) | - case management | |
| Girdler et al. (2010) | - facilitating groups - collaboration with the rehabilitation team | |
| Hauken et al. (2015) | - conducting assessments | |
| Jokel (2016) | - counseling - education - collaboration with the rehabilitation team - *social environment* | |
| Latzer (2019) | - facilitating groups - collaboration with the rehabilitation team - goal setting - aftercare activities - *disease-specific content* - *social environment* | |
| Li & Ma (2021) | - case management - engaging community resources - *work-related issues* - *self-awareness/self-care* | |
| Matthew et al. (2022) | - communication via apps - conducting telephone appointments | |
| Mengshoel & Skarbø (2017) | - *financial/social security* - *work-related issues* - *social environment* | |
| Pedersen et al. (2017) | - case management - conducting assessments - developing a rehabilitation plan - *work-related issues* - *disease-specific content* | |
| Pedersen et al. (2022) | - case management - referral to other providers - collaboration with the rehabilitation team - developing a rehabilitation plan - *work-related issues* - *disease-specific content* | |
| Precin (2011) | - facilitating groups - education - collaboration with the rehabilitation team - therapeutic activities - building a working alliance - referral to other providers - *financial/social security* - *work-related issues* - *disease-specific content* - *social environment* - *emotions, problems and conflicts* - *self-awareness/self-care* | |
| Radford et al. (2013) | - case management - coordinating care - education - collaboration with the rehabilitation team | |
| Rietberg et al. (2014) | - counseling - goal setting - *financial/social security* - *work-related issues* - *disease-specific content* - *social environment* - *emotions, problems and conflicts* | |
| Rosario et al. (2013) | - coordinating care - engaging community resources - education - collaboration with the rehabilitation team - *financial/social security* - *social environment* | |
| Rottmann et al. (2012) | - facilitating groups - work-related issues | |
| Smith et al. (2020) | - goal setting - discharge planning - *social environment* - *emotions, problems and conflicts* | |
| Stigt et al. (2013) | - conducting assessments - counseling - referral to other providers - *emotions, problems and conflicts* | |
| Ta’eed et al. (2015) | - case management - coordinating care - engaging community resources - referral to other providers - discharge planning - building a working alliance - counseling - *financial/social security* - *work-related issues* - *emotions, problems and conflicts* | |
| Young et al. (2019) | - *financial/social security* - *work-related issues* - *emotions, problems and conflicts* | |
| Yuliawiratman & Suhaimi (2015) | - *financial/social security* | |
| Zakrisson et al. (2013) | - *emotions, problems and conflicts* | |
| Zarshenas et al. (2019) | - case management - conducting assessments - discharge planning - collaboration with the rehabilitation team - education | |
